# MicroRNA-379 acts as a tumor suppressor in non-small cell lung cancer by targeting the IGF-1R-mediated AKT and ERK pathways

**DOI:** 10.3892/or.2022.8339

**Published:** 2022-05-30

**Authors:** Fangzheng Zhou, Long Nie, Dali Feng, Siyan Guo, Ren'Na Luo

Oncol Rep 38: 1857–1866, 2017; DOI: 10.3892/or.2017.5835

Following the publication of this paper, it was drawn to the Editors attention by a concerned reader that the cell migration and invasion assay data shown in Figs. 2F, 5D and 6D were strikingly similar to data appearing in different form in other articles by different authors. Owing to the fact that the contentious data in the above article had already been published elsewhere, or were already under consideration for publication, prior to its submission to *Oncology Reports*, the Editor has decided that this paper should be retracted from the Journal. After having been in contact with the authors, they agreed with the decision to retract the paper. The Editor apologizes to the readership for any inconvenience caused.

